# Behavioural and neural structure of fluent speech production deficits in aphasia

**DOI:** 10.1093/braincomms/fcac327

**Published:** 2022-12-14

**Authors:** Eleni Zevgolatakou, Melissa Thye, Daniel Mirman

**Affiliations:** Department of Psychology, University of Edinburgh, 7 George Square, Edinburgh EH8 9JZ, UK; Department of Psychology, University of Edinburgh, 7 George Square, Edinburgh EH8 9JZ, UK; Department of Psychology, University of Edinburgh, 7 George Square, Edinburgh EH8 9JZ, UK

**Keywords:** aphasia, fluency, syntax, lesion–symptom mapping, connectivity

## Abstract

Deficits in fluent speech production following left hemisphere stroke are a central concern because of their impact on patients’ lives and the insight they provide about the neural organization of language processing. Fluent speech production requires the rapid coordination of phonological, semantic, and syntactic processing, so this study examined how deficits in connected speech relate to these language sub-systems. Behavioural data (*N* = 69 participants with aphasia following left hemisphere stroke) consisted of a diverse and comprehensive set of narrative speech production measures and measures of overall severity, semantic deficits, and phonological deficits. These measures were entered into a principal component analysis with bifactor rotation—a latent structure model where each item loads on a general factor that reflects what is common among the items, and orthogonal factors that explain variance not accounted for by the general factor. Lesion data were available for 58 of the participants, and each factor score was analysed with multivariate lesion–symptom mapping. Effects of connectivity disruption were evaluated using robust regression with tract disconnection or graph theoretic measures of connectivity as predictors. The principal component analysis produced a four-factor solution that accounted for 70.6% of the variance in the data, with a general factor corresponding to the overall severity and length and complexity of speech output (complexity factor), a lexical syntax factor, and independent factors for Semantics and Phonology. Deficits in the complexity of speech output were associated with a large temporo-parietal region, similar to overall aphasia severity. The lexical syntax factor was associated with damage in a relatively small set of fronto-parietal regions which may reflect the recruitment of control systems to support retrieval and correct usage of lexical items that primarily serve a syntactic rather than semantic function. Tract-based measures of connectivity disruption were not statistically associated with the deficit scores after controlling for overall lesion volume. Language network efficiency and average clustering coefficient within the language network were significantly associated with deficit scores after controlling for overall lesion volume. These results highlight overall severity as the critical contributor to fluent speech in post-stroke aphasia, with a dissociable factor corresponding to lexical syntax.

## Teaser

Zevgolatakou *et al.* report that measures of narrative speech in post-stroke aphasia correspond to (i) general impairment severity and sentence/utterance complexity, which are associated with damage in a large temporo-parietal region and (ii) lexical syntax, which is associated with damage in a relatively small set of fronto-parietal regions.

## Graphical Abstract

**Graphical abstract:**
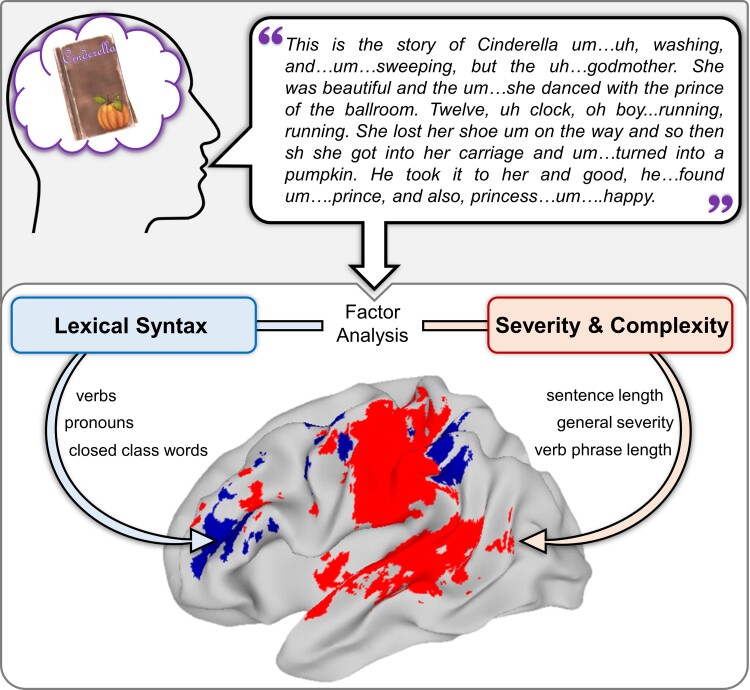

## Introduction

Aphasia is an acquired impairment of language production and comprehension that affects approximately one-third of stroke survivors and is one of the most frequent and debilitating consequences of brain injury.^1,2^ From the earliest research on aphasia,^3,4^ deficits in fluent speech production have been a central concern of both clinical and scientific importance because of their impact on patients’ lives and the insight they provide about the neural organization of language processing. In the intervening 100 + years, it has become clear that fluent speech production requires the rapid coordination of multiple cognitive and neural systems, including syntax, sentence planning and working memory, semantics, and phonology and articulatory motor control.^5-8^ This study used data-driven methods to investigate the relationships among fluency, overall aphasia severity, and deficits of syntax, semantics and phonology.

### Contributors to fluent speech production

Although aphasia is traditionally divided into ‘fluent’ and ‘non-fluent’ subtypes, this distinction has been widely criticised for not correctly capturing symptom co-occurrence and for having a poor agreement between diagnostic instruments.^9-13^ A key problem is that multiple different underlying impairments can disrupt fluent speech production. These underlying impairments reflect different cognitive sub-systems with different neural bases and may require different treatment approaches.^10^

Deficits of planning or executing the articulatory gestures that make up speech can produce hesitations, phonological errors and distortions, which are one form of non-fluent speech. Such deficits can arise from impairments in phonological planning or in articulatory motor control (e.g. dysarthria) or their interface (apraxia of speech) and are associated with damage to precentral motor control regions, postcentral somatosensory cortex, and inferior parietal regions that support phonological–articulatory planning.^7,14-16^

Difficulty with retrieving the intended word can also produce hesitations during a connected speech while the speaker searches for that word. Such difficulty results from impaired lexical–semantic processes: semantic knowledge of words and selection processes to choose among candidate words.^17,18^ Impairments of these lexical–semantic processes are associated with damage to anterior temporal and inferior frontal regions.^19-21^

Syntactic processes are also critical for fluent speech production, and there is a long history of association between ‘agrammatism’ and non-fluent aphasia.^22,23^ Agrammatism is characterized by reduced or omitted syntactic structure, which is a common characteristic of non-fluent speech. These structural elements can be at the sentence-level (e.g. well-formedness of sentences, extent of structural embeddings) or at the lexical-level (e.g. correct use of pronouns, production of determiners and closed class words). A recent comprehensive framework integrated a large body of evidence to propose that posterior superior and middle temporal regions support hierarchical lexical–syntactic functions for production and comprehension while inferior frontal regions support morpho-syntactic sequencing primarily relevant for production.^6^ This view was supported by a dissociation between reduction/omission of grammatical structure (‘agrammatism’), which was associated with frontal damage, and grammatical errors that were not reductions (‘paragrammatism’), which was associated with posterior superior and middle temporal damage.^24^

However, the neural basis for syntactic processing is somewhat inconsistent across studies, implicating large regions of frontal, parietal, and temporal cortex, as well as the underlying white matter. Some evidence suggests that the language network shows lexical–semantic and combinatorial sensitivity with no regions selective for purely syntactic processes.^25^ In contrast to a syntax-specific view, some have argued that syntactic deficits are a result of relatively general cognitive resource reductions.^26^ At least one study^7^ found no regions where damage was associated with a syntactic deficit; it is likely that other such null results exist but have been suppressed by publication bias.

Thus, fluency deficits could plausibly arise from phonological/articulatory, lexical–semantic, or syntactic deficits. Connectivity within these systems also plays an important role: several studies have found that damage to frontal and peri-Sylvian white matter tracts is an important contributor to fluency deficits.^27-30^ More recent ‘network neuroscience’ work has quantified connectivity disruption using graph theory measures, which are meant to capture broader connectivity properties like the efficiency of information transmission and integration or segregation of neural components.^31^ One study found that graph theory measures outperformed connection weights as predictors of fluency deficits.^32^

A further complication is that recent studies have identified aphasia severity as a major dividing feature. A large-scale study of 330 participants with aphasia across three different aetiologies (post-stroke aphasia, primary progressive aphasia, and post-operative aphasia) found that severity was the primary dimension of variability, explaining 75% of the variance in aphasia battery sub-scores.^33^ A more focused examination of 226 participants with post-stroke aphasia found that a ‘mild aphasia’ cluster was behaviourally and neuroanatomically distinct from the other two clusters, which corresponded to the semantic deficit and phonological deficit.^34^

### Multi-dimensional and data-driven approaches to fluency

Data-driven statistical methods like factor analysis and principal component analysis (PCA) use correlations among measures to group them into latent factors or components and calculate composite scores for those factors. Several studies have focused on measures of connected speech and used factor analysis to identify clusters of measures that suggest common underlying components^5,35-37^ (for recent reviews of factor analysis approaches in contemporary aphasia research see^38,39^). A key factor analysis study^5^ of spontaneous speech in 274 individuals with aphasia identified 6 latent factors that accounted for 52% of the variance, with substantial overlap between some of the factors. In particular, four of the factors were strongly correlated and primarily reflected fluency (words per minute) and syntactic structure (utterance length, use of grammatically complex structures, propositional density, verb marking and grammatical errors). The other factors reflected semantic anomalies (e.g. jargon) and utterance repairs.

Because multiple cognitive sub-systems contribute to fluent speech production, it is important to consider them simultaneously. That is, combining measures derived from connected speech elicitation tasks with other measures that capture specific sub-systems is necessary for investigating how fluency relates to those sub-systems. A study of 50 participants with primary progressive aphasia^40^ found that frontal regions were associated with speech sound distortions and syntactic deficits, anterior and inferior temporal regions with lexical retrieval, and posterior temporal regions with phonological errors. In post-stroke aphasia, a statistical path modelling analysis^8^ found that syntactic impairment had a strong and direct association with fluency, while word production, comprehension and working memory deficits were indirectly associated with fluency.

Such multi-dimensional approaches can allow investigation of how the sub-systems relate to one another in addition to how they contribute to fluency. This is particularly important for the domain of syntax deficits, which (as briefly reviewed in the previous section) have sentence-level and word-level aspects, and could result from general resource reduction (which can be reflected in overall severity) or combinatorial deficits rather than syntax-specific impairments.

Several recent studies have applied this approach and identified consistent dissociations between fluency, semantic, phonological, and executive deficits.^39^ In these studies, the fluency components had high loadings from composite measures of spontaneous speech production [e.g. fluency scores from aphasia assessments such as the Western Aphasia Battery (WAB) and Comprehensive Aphasia Test] and coarse measures of fluency (such as words per minute and mean length of utterance). A recent study^41^ used finer-grained discourse production measures and showed that connected speech production had distinct components of quantity (total number of content words and number of unique content words), quality (ratio of total words to unique words, also known as the type–token ratio), and words per minute (which they labelled ‘motor speech ability’). Of note, these studies included varied measures of fluency but very limited measures of syntax. Another recent study specifically focused on connected speech production in acute left hemisphere stroke^42^ and found distinct components of utterance length/complexity, syntactic accuracy, lexical retrieval, and words per minute (which they labelled ‘production fluency’). This last study suggests dissociations between phonological, semantic, syntactic, and combinatorial processes, but the semantic measures were limited, and phonological measures were virtually absent, so the connection to those broader components remains unclear.

### The current study

The current study used a data-driven approach to evaluate how measures of fluency and syntax deficits in post-stroke aphasia relate to overall aphasia severity, semantic deficits, and phonological deficits, and the lesion correlates of these components. We build on prior data-driven work, making several key innovations and improvements. First, we used quantitative production analysis (QPA)^23^ to derive a diverse set of narrative speech production measures that include fluency and syntax at lexical, utterance and sentence levels. Like Ding et al.,^42^ we used a comprehensive set of QPA measures; however, we also included general measures of language impairment and measures of semantic and phonological deficits. These additional measures served as ‘anchors’ for three well-established aspects of aphasic deficits (overall severity, semantic deficit, and phonological deficit) allowing the QPA-based measures of fluency and syntax deficit to attach to those factors or to form separate factors.

Second, to explicitly capture overall severity as a core dimension of variability in aphasia, we used a bifactor PCA model. Bifactor modelling re-emerged relatively recently as a psychometric technique that hypothesizes a single general factor, which explains shared variance across all items (measures), and a series of orthogonal (uncorrelated) domain-specific factors.^43,44^ Because we included anchor variables and used bifactor rotation, we expected to find severity, semantic, and phonological factors; the key research question was how the QPA-based measures of fluency and syntax would distribute among those factors or form additional factors.

Third, we conducted lesion–symptom mapping (LSM) analyses using a multivariate optimization technique known as sparse canonical correlation analysis for neuroimaging (SCCAN), which more accurately identifies symptom-relevant lesion areas.^45^ SCCAN is particularly important for the present study because fluency and syntax are likely to rely on a distributed network of brain regions, and SCCAN is better able to detect networks of symptom-relevant brain regions.^45^ In addition, SCCAN uses cross-validation (CV) to optimize the sparseness of the solution and evaluates the prediction accuracy (and the statistical significance) of the overall solution. As a result, the accuracy of lesion-deficit prediction determines the size of the critical region identified by the LSM analysis. In contrast, in mass-univariate LSM, the focality of the result is based on the conservativeness of the correction for multiple comparisons, which is set based on statistical considerations.^46-48^ Therefore, SCCAN is better able to capture distributed networks of lesion-relevant regions and to determine whether there are (possibly multiple) small symptom-relevant regions or a single large region.

Fourth, based on the premise that connectivity disruption can undermine functional networks that support language production, we tested the contribution of white matter damage using both tract-based and network-based measures. This was motivated by prior studies that highlighted the role of white matter damage [particularly the frontal aslant tract (FAT) and the anterior portion of the arcuate fasciculus (AF)]^27,49,50^ in fluency and syntax deficits, although we did not find specific effects of frontal white matter damage in our prior work.^7^ That is, the current state of the evidence is equivocal, possibly because there is substantial variability in both the methods and results of prior work on effects of connectivity disruption on language deficits in post-stroke aphasia. Therefore, these exploratory analyses were intended to help build toward a consensus rather than to test specific hypotheses. In the current study, we evaluated the effects of damage to a larger set of language-relevant white matter tracts [left AF, superior longitudinal fasciculus, inferior fronto-occipital fasciculus (IFOF), uncinate fasciculus (UF), and FAT]. We also evaluated the effects of connectivity disruption using measures derived from graph theory (global efficiency, characteristic path length, average clustering coefficient), which are meant to capture broader communication efficacy and efficiency.

In sum, the present study provides a comprehensive behavioural and neural assessment of fluency deficits in post-stroke aphasia. A data-driven approach was used to determine how a diverse set of measures of fluency and syntax processing were associated with or dissociated from measures of aphasia severity, semantic deficits, and phonological deficits. Multivariate LSM and connectivity disruption analyses were then used to determine the neural correlates of these behavioural deficit dimensions.

## Materials and methods

### Participants

The initial data set consisted of behavioural data from 74 participants who were native English speakers, right-handed, and had suffered a left hemisphere stroke. These participants are the subset who completed the connected speech elicitation task from a larger study of post-stroke aphasia.^51^ We conducted a preliminary leave-one-out PCA stability analysis in which we iteratively removed an individual participant and tested the effect of that exclusion on the PCA results (this is a non-parametric version of standard outlier detection methods). In general, there was a very high correlation (>0.95) between PCA results with and without a participant, indicating stable results that are minimally influenced by any one participant. However, there were five high-influence participants: excluding one of these participants substantially changed the results. These five outlier participants were excluded from the analysis (these were multivariate outlier cases: their pattern of performance did not conform to the factor structure that described the rest of the participants. However, they were not outliers on any one dimension and their performance profiles were not particularly similar to one another. We speculate that these participants were using idiosyncratic strategies so their performance on some measures was substantially better than would be expected based on their performance on other measures). The analytic sample contained behavioural data from 69 participants and imaging data were available for 58 of these participants.

All but five participants were examined at the chronic stage of aphasia (at least 3 months post-onset), but the exclusion of these five participants did not change the results so the more inclusive analyses are reported here. The data are part of a larger study on language processing following left hemisphere stroke and several previous articles on language deficits in aphasia have included subsets of the participants.^7,19,34,48,52,53^ A summary of demographic and clinical information is shown in Table 1. The original data collection and sharing were approved by Institutional Review Boards at the Einstein Healthcare Network and University of Pennsylvania School of Medicine. The analyses of de-identified data in the current study were approved by the University of Edinburgh PPLS Research Ethics Committee.

**Table 1:** Participant demographic and clinical information for the participants included in the behavioural analysis (N = 69) and the subset who had lesion data (N = 58)

Variable	Behavioural analysis sample (N = 69)	Lesion analysis sample (N = 58)
Sex (F:M)	33:36	27:31
Age	58.0 (50.0–68.0) [31.0–79.0]	58.5 (51.3–68.0) [31.0–79.0]
Months post-onset	23.0 (7.0–75.0) [0.5–234]	28.0 (7.0–81.0) [0.5–234]
Education (years)	14.0 (12.0–18.0) [10.0–21.0]	14.0 (12.0–18.0) [10.0–21.0]
WAB AQ	83.1 (71.7–90.4) [47.2–99.3]	81.3 (71.3–91.0) [47.2–99.3]
WAB fluency	8.0 (5.0–9.0) [2.0–10.0]	8.0 (5.0–9.0) [2.0–10.0]
Total words	231.0 (202.0–264.0) [99.0–404.0]	230.0 (201.2–263.4) [99.0–404.0]
Words per minute	64.5 (44.8–86.6) [14.1–193.5]	61.6 (43.3–88.5) [14.1–193.5]
Med Utter Len	5.0 (4.0–6.0) [1.0–11.0]	5.0 (4.0–6.0) [1.0–8.5]
PNT: Correct	0.79 (0.65–0.86) [0.23–0.97]	0.79 (0.65–0.86) [0.23–0.97]
PNT: Semantic Err	0.034 (0.017–0.051) [0–0.11]	0.034 (0.017–0.051) [0–0.11]
PNT: Formal/NW Err	0.068 (0.04–0.16) [0.006–0.37]	0.068 (0.042–0.17) [0.006–0.37]
PRT	94.0 (87.0–97.0) [57.0–100.0]	94.0 (86.3–96.8) [57.0–100.0]
Camel and Cactus	80.0 (72.0–84.0) [45.0–94.0]	80.0 (72.0–83.8) [45–94]
Semantic Discrimination	90.0 (85.0–93.0) [65.0–100.0]	89.0 (85.0–93.0) [65.0–100.0]
Non-word repetition	52.0 (32.0–70.0) [8.0–95.0]	53.5 (30.5–72.3) [8.0–95.0]
Aphasia classification		
Anomic	40	31
Broca	13	14
Conduction	10	9
TSA	2	2
TMA	3	1
Wernicke	1	1

Note: Values are number of participants or medians with IQR in parentheses and range in square brackets.

Err = error, NW = non-word, TSA = transcortical sensory aphasia, TMA = transcortical motor aphasia, Med Utter Len = median utterance length.

### Image acquisition and preprocessing

Lesion location was assessed based on MRI (*N* = 45) or CT (*N* = 13) brain scans, following the same procedures as previous studies of this data set.^7,19,34,48,52,53^ For the MRI scans, lesions were manually segmented on each participant’s T1-weighted structural image by a technician and reviewed by an experienced neurologist for accuracy. Each participant’s brain image was registered to the Montreal Neurological Institute space Colin27 template by an automated symmetric diffeomorphic registration algorithm,^54^ which iteratively samples the space to find the best solution to align the intact portion of the brain (i.e. with the lesion masked out) with the template. This solution (image transformation) is then applied to the lesion mask to register it to the same template. For the CT scans, a neurologist drew the lesions directly onto the Colin27 template after rotating it (pitch only) to match the approximate slice plane of the participant’s scan.

### Quantitative production analysis

Connected speech samples were primarily elicited via the ‘Cinderella story’ with the requirement that every elicited narrative had at least 150 words; if necessary, additional connected speech was elicited using other well-known stories (such as ‘The Little Red Riding Hood’) in order to reach at least 150 words. The speech samples were transcribed and coded by a speech pathologist or research assistant specifically trained to perform transcription and QPA coding following guidelines based on published QPA scoring protocol.^23,55,56^ The protocol is designed to characterize aphasic sentence production, focusing on syntax and more general measures of fluency, and has been adapted to accommodate fluent as well as non-fluent participants. During training, all coders achieved ∼90% agreement on their transcription and coding of utterance boundaries, utterance content and grammatical structure.

The measures selected for the analyses reflected a wide range of morphological, structural and lexical properties of speech. Based on the grouping described in the QPA manual,^56^ the measures correspond to lexical content (words per minute, proportion of closed class words, proportion of pronouns, proportion of verbs, determiner index, and inflection index), auxiliary verb usage (auxiliary complexity index), and structural analysis [proportion of words in sentences, proportion of well-formed sentences, embedding index, mean sentence length, median utterance length, mean verb phrase (VP) length]. See Supplementary Table 1 for an additional explanation of the QPA measures. This QPA manual grouping was used as a starting point, but the grouping and characterization of these measures differ somewhat across studies,^23,55,57^ and a goal of this study was to evaluate how these measures relate to aphasia severity, semantic deficits and phonological deficits.

### Additional behavioural assessments

The behavioural testing of the participants consisted of a wide range of neuropsychological batteries and tests. To supplement the QPA measures, a subset of measures was selected to reflect overall severity and two important contributors to language production that are not captured by QPA measures: phonology and semantics. These measures provide are optimized for capturing phonological and semantic deficits and are independent of connected speech, so they provide a stronger assessment of whether aspects of connected speech are a result of such deficits.

1. *WAB-Revised*:^58^ A standard aphasia assessment battery that provides an overall measure of aphasia severity—the aphasia quotient (AQ). The measure used is WAB AQ.
2. *Philadelphia Naming Test* (PNT):^59^ A test of single word production in object (picture) naming that provides measures of overall word production deficit (accuracy) as well as semantic and phonological deficits (semantic and phonological errors, respectively). Measures used are as follows: proportion of correct responses, proportion of semantic errors and proportion of formal and non-word errors.
3. *Camel and Cactus Test* (CCT):^60^ A picture-based semantic association test. The measure used is percentage of correct responses.
4. *Semantic discrimination*: A verbal semantic judgment task derived from the semantic category probe task^61^ by using only list-length 1 data. The measure used is percentage of correct responses.
5. *Philadelphia Repetition Test* (PRT):^62^ A test of word repetition using the same words as the PNT. This test primarily measures phonological processing with lexical contributions.^63,64^ The measure used is percentage of correct responses.
6. *Non-word repetition task*:^61^ A test of non-word repetition, which primarily measures phonological production. The measure used is percentage of correct responses.

### Principal component analysis

The 21 behavioural measures (13 from QPA, 8 additional measures) were entered into a principal component analysis (PCA) with a bifactor rotation. (Analogous exploratory factor analysis produced virtually identical factor loadings [*r* > 0.98 for each of the four factors/components] and it is more straightforward to compute individual participant scores from PCA, so PCA was used here). A bifactor model is a latent structure model where each item loads on a general factor that reflects what is common among the items, and two or more orthogonal factors that potentially explain variance not accounted for by the general factor.^44^ In this case, overall aphasia severity plausibly affects all measures, so using a bifactor model explicitly allows all measures to load on this general factor. The measures that load strongly and only on this factor will be ones that are very closely related to aphasia severity. The additional factors can then represent language sub-systems that may be affected by stroke independently of overall severity. In other words, the bifactor model allows directly exploring the extent to which items reflect a common trait (aphasia severity) and the extent to which they reflect statistically independent sub-systems (semantics, phonology, syntax, etc.). The resulting component scores for every participant were then used for the LSM analysis.

### Lesion–symptom mapping analysis

LSM analyses were performed using SCCAN.^45,65^ SCCAN is an optimization algorithm that finds a set of weights that maximize the relationship between behavioural scores and voxel lesion values. Like other multivariate LSM methods, SCCAN considers all voxels together rather than individually. In addition, voxel weights are smoothed and very small clusters of voxels are set to zero to avoid the inclusion of isolated voxels in the solution. Most importantly, the SCCAN LSM result is governed by a ‘sparseness’ value that defines the proportion of voxels that are included in the solution. CV is used to empirically determine an optimal sparseness value that maximizes the predictive accuracy of the solution (i.e. maximum correlation between observed and predicted behavioural scores) while minimizing the number of voxels in the solution (i.e. sparser solutions are favoured over less sparse ones). As a result, if a behavioural score can be predicted from lesion in a small subset of voxels, SCCAN will identify this subset of voxels; if damage throughout a large territory is associated with the behavioural score, then SCCAN will find that non-sparse solution.

The goodness of the SCCAN solution is the correlation between the predicted and observed behavioural scores and the statistical significance of that correlation is used to determine whether the SCCAN solution is acceptable. If SCCAN is not able to identify a set of voxels that statistically significantly predict the behavioural score, then SCCAN has failed to identify an adequate solution. Critically, the SCCAN solution is evaluated in terms of its overall predictive accuracy, not individual voxels within it, so there is only one overall statistical test, not individual tests for voxels. This is in stark contrast to mass-univariate LSM and some multivariate LSM methods (such as LSM using support vector regression^66^), where statistical significance is evaluated at the level of individual voxels and requires correcting for multiple comparisons across many voxels. The conservativeness of this multiple comparisons correction has a major impact on the focality of results. Unlike these other LSM methods, the sparseness or focality of SCCAN LSM results is determined by predictive accuracy of the overall solution.

### Measures of connectivity disruption

Connectivity disruption measures were derived using atlas-based metrics of lesion impact generated in MATLAB 2020b^67^ using the Lesion Quantification Toolkit^68^ and DSI Studio (version date: Oct 24, 2020; http://dsi-studio.labsolver.org). The toolkit calculates connectivity disruption by overlaying the lesion files on an atlas of intact connectivity.^69^ This whole-brain connectivity atlas was derived using deterministic tractography on the population-averaged HCP-842 data set with multiple turning angle thresholds to obtain 500 000 population-level streamline trajectories (i.e. estimated white matter fibre trajectories based on directional diffusion information), which were manually vetted and assigned to known white matter fibre tracts by a team of neuroanatomists.^69^

Participant binarized and spatially normalized lesion files were used as inputs to the toolkit, which was run with the following parameters: connection criteria = pass, spared connection threshold = 50 and smoothing kernel = 2. The primary atlas-based measures of interest were (i) disconnection severity within critical left hemisphere white matter tracts, (ii) metrics describing the whole-brain network structure, and (iii) metrics describing the regional structure of a language sub-network.

Tract-based disconnection severity was calculated by overlaying the lesion files on each tract within the HCP-842 tractography atlas^69^ and quantifying the number of streamlines intersecting the lesion relative to the total number of streamlines within the tract. This method of estimating tract disconnection severity has been shown to be more sensitive than traditional lesion load measures.^70^ Disconnection severity within the left superior longitudinal fasciculus (SLF), left AF, left UF, left FAT, and left IFOF were used for analyses.

In order to generate network metrics, a structural connectivity matrix was constructed for each participant by starting with the streamline trajectories included in the HCP-842 tractography atlas,^69^ removing streamlines that intersected with the participant’s lesion, and counting the number of remaining streamlines that passed through each pair of regions within the AAL3 atlas.^71^ The standard implementation of the Lesion Quantification Toolkit outputs *disconnection* severity matrices (i.e. the number of streamlines that intersect the lesion territory). To compute a *connectivity* matrix, the number of preserved streamlines was estimated as the number of streamlines within the HCP-842 tractography atlas that did *not* intersect with the lesion territory. Graph theoretical measures quantifying the network-level impact of the lesion were derived using DSI Studio’s implementation of the Brain Connectivity Toolbox^31^ applied to each participant’s structural connectivity matrix where each element in the matrix corresponds to the count of preserved streamline connections between a pair of regions.

Structural connectivity matrices and corresponding graph theoretical measures were also generated for a language sub-network comprised of the following left hemisphere regions within the AAL3 atlas and based on prior work using similar methods^32,^  ^72-76^ middle frontal gyrus, inferior frontal gyrus (IFG) (pars opercularis, pars triangularis, and pars orbitalis), precentral gyrus, postcentral gyrus, insula, superior portion of the temporal pole, middle portion of the temporal pole, inferior temporal gyrus, middle temporal gyrus, superior temporal gyrus, supramarginal gyrus (SMG) and angular gyrus. The global, weighted network measures for the whole brain and the regional language network were used for subsequent analyses.

**Figure 1:**
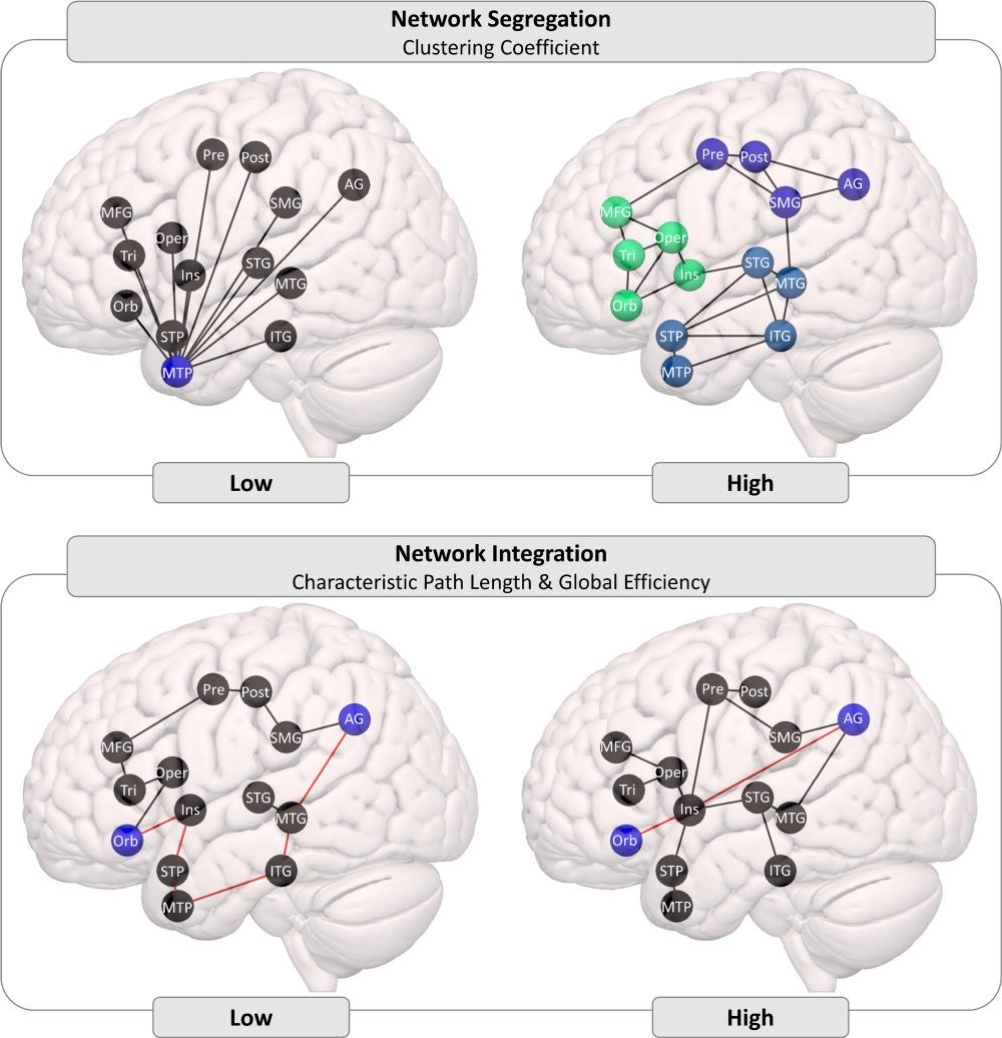
**Schematic illustrations of connectivity differences derived from graph theory**. Top: high clustering coefficient (shown on the right) indicates more densely interconnected sub-networks than low clustering coefficient (shown on the left). Bottom: high network integration indicates that, on average, fewer steps are needed for information to get from one node to any other node. The AG-Orb path is an illustrative example: in the low integration case (shown on the left), a minimum of six steps is required, in the high integration case (shown on the right), only two steps are required. Note: these are schematic illustrations to clarify the graph theoretic measures of connectivity, they do not reflect claims about patterns of connectivity among specific brain regions.

Three graph theoretical measures were selected for analyses (and are illustrated in Fig. 1):

1. *Average clustering coefficient*: the average of the clustering coefficients of every node within a network. Clustering coefficient is a measure of network segregation, capturing the extent to which functionally related regions are densely connected into specialized clusters. Damage within a functionally segregated, densely connected cluster can impact the efficiency of local processing^77,78^ but may have a limited effect on other clusters, thus allowing other functions to be preserved.
2. *Characteristic path length*: the average of the shortest paths between all pairs of nodes within the network. The characteristic path length is a measure of network integration based on how many steps are required to get from one node to another node. Higher values indicate that (on average) information must travel through more steps to get from one node to another, suggesting a less functionally integrated network.^31^ It is associated with reduced functional connectivity,^79^ degree of language impairment,^32,68^ and fluency in particular^32^ (some studies refer to path length as ‘propagation speed’ or ‘propagation steps’).
3. *Global Efficiency*: the average of each individual node’s global efficiency, which is calculated by starting with the shortest path lengths between that node and all other nodes (as for characteristic path length), taking the inverse of those path lengths, and averaging those values. Like characteristic path length, global efficiency is a measure of network integration that captures how quickly information can travel between brain regions,^80^ but the difference in their calculation has substantial implications. If two nodes are not connected, the path length between them is infinite and the communication efficiency is 0. When calculating the average, these values have a radically different impact (i.e. disconnected nodes very strongly increase characteristic path length, but only moderately decrease global efficiency). More generally, the characteristic path length is strongly influenced by long paths (infinite path length between disconnected nodes is an extreme case) but efficiency is more sensitive to short paths. This difference may be further exaggerated when these measures are calculated for stroke-damaged brains, which are more likely to have disconnected nodes or to rely on longer paths to communicate around the lesion territory. Johnson et al.^77^ found that higher global efficiency within the semantic network was associated with better response to naming therapy in chronic post-stroke aphasia.

### Statistical analysis

PCA was implemented using the psych package version 1.9.12,^81^ with the bifactor rotation implementation from Jennrich and Bentler.^82^ The behavioural measures were checked for factorability with a Kaiser–Meyer–Olkin (KMO) test; conventionally, KMO > 0.7 is considered adequate for factor analysis and KMO < 0.5 is unacceptable. A scree plot, combined with a parallel analysis and Velicer's minimum average partial (MAP) test, was used to determine the number of dimensions that should be retained.

The SCCAN LSM analysis was conducted in R version 3.6 using the package LESYMAP version 0.0.0.9221 (https://github.com/dorianps/LESYMAP). SCCAN LSM was run separately for each of the factors that resulted from the PCA (and a supplementary analysis for WAB AQ), with sparseness optimized independently for each analysis using 8-fold CV (the number of folds was increased from the default 4-fold CV to improve stability of the results). The LSM solution was evaluated based on the correlation between predicted and observed behavioural scores. This evaluation applies to the overall LSM solution, not to individual voxels within the solution, so it is only one test and does not require correction for multiple comparisons.

Associations between connectivity measures and behavioural deficits were tested first using bivariate correlations, then multiple regression to assess simultaneous impact of multiple types of connectivity damage. Separate regressions were run for each behavioural deficit type and connectivity damage group (tracts, network metrics). Several of the connectivity measures had skewed or bimodal distributions, so spearman rank correlations were used for bivariate correlations and multiple regression analyses used robust standard error estimation^83^ implemented in the lavaan package version 0.6–8.^84^ Results are reported as coefficient estimates and 95% confidence intervals (95% CIs).

## Results

### Principal component analysis

Bivariate correlations among the behavioural measures are shown in Supplementary Figure 1. The behavioural measures had adequate overall factorability (KMO = 0.78) and no individual measure was in the ‘unacceptable’ range (all KMO > 0.5). Scree plot, parallel analysis, and MAP test all suggested the extraction of four factors.

**Figure 2:**
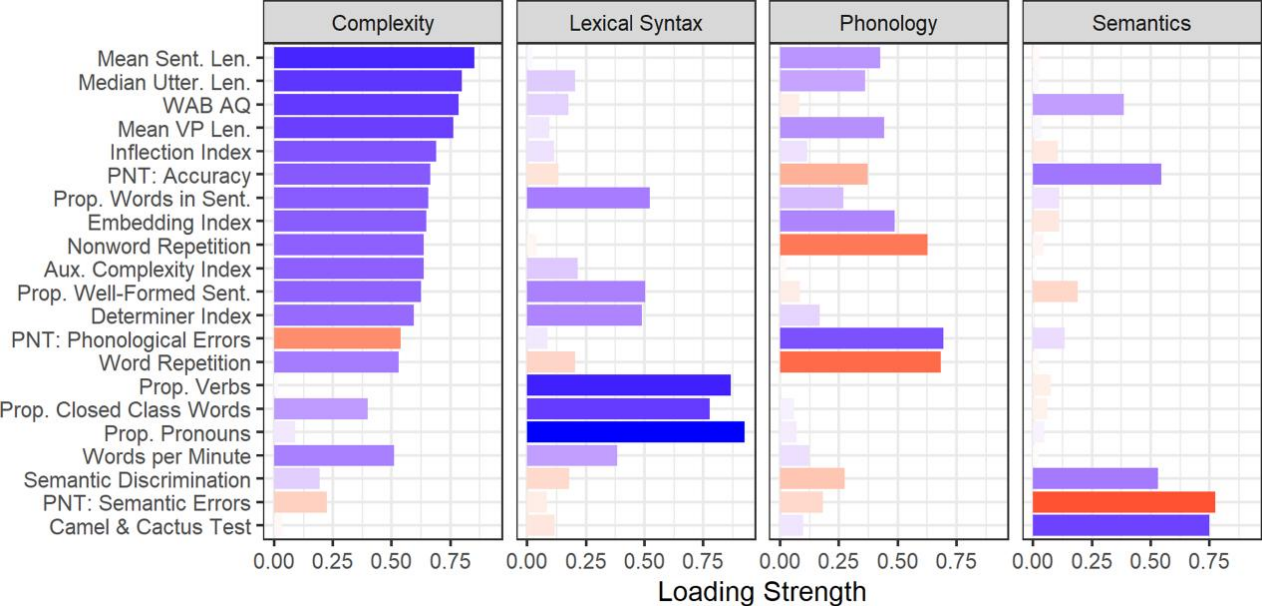
**Bifactor PCA factor loadings**. Each panel shows a single factor. Bar length and saturation indicate absolute loading magnitude. Colour indicates direction of the loading: positive loadings are blue, negative loadings are red. Note: Sent = Sentence(s), Len = Length, Utter = Utterance, VP = Verb Phrase, Prop = Proportion, Aux = Auxiliary.

Factor loadings from a four-factor PCA with bifactor rotation are shown in Fig. 2 (and in Supplementary Table 2). These four components accounted for 70.6% of the variance (RC1: 33.1%, RC2: 16.1%, RC4: 11.9%, RC3: 9.5%). By design, the first principal component reflected the general underlying factor resulting from the bifactor rotation. As expected, it had high positive loadings from general severity measures (WAB AQ and PNT accuracy). In addition, it contained high positive factor loadings from measures of utterance/sentence length (mean sentence length, median utterance length, mean VP length) and measures of sentence structural integrity and complexity (proportion of words in sentences, inflection index, embedding index). This suggests that the quantity (length) and complexity of speech output is closely related to overall severity, while other aspects can be more effectively separated from severity. We will refer to this factor as ‘complexity’ because that is the aspect of fluent speech production that was uniquely associated with this factor, although it also reflects general aphasia severity.

The second factor had high positive loadings of lexical syntax measures that are considered critical for detecting agrammatic speech production (proportions of closed class words, verbs and pronouns) and moderate loadings from sentence planning measures (proportion of words in sentences, proportion of well-formed sentences). We will refer to this factor as ‘lexical syntax’. The third factor had high negative loadings from tests of repetition of words (PRT) and non-words (non-word repetition task) and a high positive loading from the proportion of phonological errors in picture naming, so it was named ‘Phonology’ (note that this means that a participant’s factor scores for this factor are reversed relative to the other factors: for other factors, higher scores indicate better performance, but for Phonology, higher scores indicate a more severe phonological *deficit*). The last factor was labelled ‘Semantics’ because it had high negative loadings from the proportion of semantic errors from the PNT and high positive loadings on the percentage of correct responses from the CCT and the semantic discrimination task.

In addition to the overall factor structure, the data-driven PCA approach yielded non-obvious observations about individual measures, such as the inflection index, determiner index and use of pronouns. Inflections are syntactic elements, and their omission is associated with agrammatism,^85^ so one might have expected them to be part of a syntax factor. Inflections in English typically add phonological complexity (e.g. plural/-s/and past-tense/-d/or/-t/), so it is also possible that their omission is a result of a phonological/articulatory deficit and would cluster with other measures of phonological production deficits. Neither of these turned out to be the case in the present data: the inflection index measure clustered with general measures of aphasia severity (WAB AQ), fluency (words per minute), and sentence and utterance length. Determiners are also small phonological elements that primarily serve a syntactic function, so one might expect them to pattern with inflections. Like inflection index, determiner index did strongly load on the first factor, but it also strongly loaded on the second (lexical syntax) factor. In contrast, use of pronouns—which are short and primarily serve a structural role—loaded almost exclusively on the lexical syntax factor (with verbs and closed class words), not on the first factor or on the Phonological factor. This pattern highlights that the ‘lexical syntax’ factor appears to be specific to production of *lexical* elements that primarily serve a syntactic (rather than semantic) function more so than other aspects of syntax.

### Sparse canonical correlation analysis

**Figure 3:**
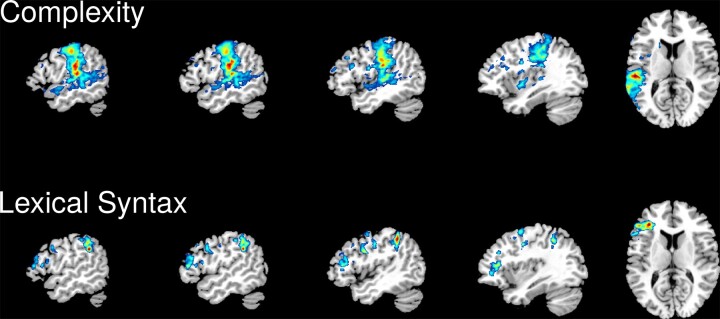
**SCCAN LSM results.** Complexity of speech output (top row) and lexical syntax (bottom row). The colours correspond to normalized SCCAN weights in the range 0–1. All results are shown on the same slices of an MNI template (from left to right: x = −53, −50, −45, −36 and z = 11).

Lesion coverage was good throughout the left middle cerebral artery (MCA) territory, particularly the dorsal speech production system structures of the frontal lobe and inferior parietal lobe (Supplementary Figure 2). SCCAN LSM results are shown in Fig. 3 and Table 2.

**Table 2:** Proportion of each AAL region implicated for SCCAN LSM analyses of complexity and lexical syntax scores

Brain region	Complexity	Lexical syntax
Inferior parietal lobule	0.301	0.231
Supramarginal gyrus	0.660	0.063
Angular gyrus	0.001	0.029
Postcentral gyrus	0.454	0.020
Precentral gyrus	0.067	0.124
Inferior frontal gyrus, pars triangularis	0.065	0.195
Inferior frontal gyrus, pars opercularis	0.010	0.035
Middle frontal gyrus	0.048	0.052
Superior frontal gyrus	0.003	0.012
Insula	0.097	0.003
Superior parietal gyrus	0.052	0.002
Middle occipital gyrus	0.003	0.0
Superior temporal gyrus	0.612	0.0
Middle temporal gyrus	0.278	0.0
Heschl’s gyrus	0.271	0.0
Rolandic operculum	0.219	0.0
Caudate	0.051	0.0
Superior temporal pole	0.031	0.0
Putamen	0.005	0.0

Note. All regions refer to the left hemisphere.

Deficits in complexity of speech output were associated with a large temporo-parietal region (optimal sparseness = 0.576, CV correlation = 0.603, *P* < 0.001): SMG and postcentral gyrus, posterior superior temporal gyrus and middle temporal gyrus, and Heschl’s gyrus (Fig. 3, top row). This result is very similar to the pattern we have previously observed for overall aphasia severity (WAB ) and replicated in this sample (see supplemental materials).

Deficits on the lexical syntax factor were associated with damage in a relatively small set of fronto-parietal regions (optimal sparseness = 0.216, CV correlation = 0.517, *P* < 0.001): IFG pars triangularis, precentral gyrus, and the dorsal portion of the inferior parietal lobule (Fig. 3, bottom row). SCCAN LSM analyses for the Phonology and Semantics factors did not produce statistically significant solutions as indicated by low CV correlations (Phonology CV correlation = 0.085, *P* = 0.524; Semantic CV correlation = 0.185, *P* = 0.164).

### Connectome analyses

The behavioural scores tended to be more strongly correlated with lesion volume than with connectivity measures (see Supplementary Table 3). Therefore, the results reported here are based on multiple regression analyses that control for lesion volume. In multiple regression analyses with lesion volume and damage to each of the tracts as predictors, lesion volume was a significant predictor of complexity [*Estimate* = −0.006, 95% CI = (−0.009, −0.003), *P* = 0.030], lexical syntax [*Estimate* = −0.007, 95% CI = (−0.011, −0.002), *P* = 0.005] and semantics [*Estimate* = −0. 005, 95% CI = (−0.009, 0.001), *P* = 0.010] but not phonology [*Estimate* = 0.000, 95% CI = (−0.003, 0.004), *P* = 0.905]. None of the tracts had statistically significant associations; full regression results are reported in Table 3.

**Table 3:** Results of multiple regression with robust estimation of standard errors for connectivity disruption predictors of each deficit measure

	Complexity	Lexical syntax	Phonology	Semantics
Tracts				
Volume	−0.0063 (−0.0095, −0.0032)**	−0.0066 (−0.011, −0.002)**	0.00021 (−0.0032, 0.0036)	−0.0048 (−0.0085, −0.0011)*
Arcuate fasciculus	0.008 (−0.0021, 0.018)	−0.0068 (−0.014, 0.00049).	0.0097 (−0.0027, 0.022)	0.00068 (−0.0069, 0.0083)
Frontal aslant tract	−0.0017 (−0.0091, 0.0056)	−0.0014 (−0.0078, 0.005)	0.0012 (−0.0069, 0.0093)	−0.0054 (−0.014, 0.0036)
Superior longitudinal	−0.0081 (−0.019, 0.003)	0.00041 (−0.0099, 0.011)	−0.0095 (−0.022, 0.0033)	0.01 (−0.0015, 0.022).
Uncinate fasciculus	0.0044 (−0.0056, 0.014)	0.0025 (−0.0093, 0.014)	−0.005 (−0.017, 0.0068)	0.0053 (−0.0064, 0.017)
Inferior fronto-occipital	0.000039 (−0.008, 0.0081)	0.0047 (−0.0031, 0.012)	−0.0029 (−0.013, 0.0072)	−0.0057 (−0.015, 0.0038)
Whole-brain network				
Volume	−0.0072 (−0.011, −0.0037)**	−0.0089 (−0.014, −0.0034)**	0.00011 (−0.0042, 0.0044)	−0.0062 (−0.01, −0.002)**
Global efficiency	−13 (−200, 170)	−150 (−380, 81)	130 (−82, 350)	−61 (−270, 150)
Ave clustering coefficient	150 (−390, 700)	340 (−300, 980)	−340 (−940, 260)	490 (−46, 1000).
Characteristic path length	0.00070 (−0.0013, 0.0027)	−0.0012 (−0.0035, 0.0012)	0.00022 (−0.0022, 0.0027)	0.0029 (0.00051, 0.0054)*
Language network				
Volume	−0.0072 (−0.0097, −0.0046)**	−0.0078 (−0.011, −0.0045)**	−0.00011 (−0.0026, 0.0024)	−0.0025 (−0.0059, 0.00096)
Global efficiency	1.4 (0.084, 2.8)*	2.8 (1.6, 4)**	−2.1 (−3.2, −0.91)**	−2 (−3.3, −0.65)**
Ave clustering coefficient	−2.1 (−13, 8.8)	8.8 (−0.5, 18) .	−8.1 (−16, −0.54)*	9 (1.2, 17)*
Characteristic path length	−0.00011 (−0.0015, 0.0013)	0.00028 (−0.00084, 0.0014)	−0.0012 (−0.0023, −9.8e-05)*	−0.00019 (−0.0011, 0.00077)

Note: Values show the regression coefficient estimate (95% confidence interval in brackets).

** *P* < 0.01, * *P* < 0.05, *P* < 0.1.

In analogous analyses of the graph theoretical measures of connectivity, the same lesion volume effects were statistically significant. In addition, language network efficiency was a significant predictor for each of the four PCA factors: Complexity [*Estimate* = 1.424, 95% CI = (0.084, 2.765), *P* = 0.037], Lexical syntax [*Estimate* = 2.781, 95% CI = (1.572, 3.989), *P* < 0.001], Phonology [*Estimate* = −2.064, 95% CI = (−3.215, −0.913), *P* < 0.001] and Semantics [*Estimate* = −1.986, 95% CI = (−3.326, −0.645), *P* = 0.004]. Language network average clustering coefficient was also a significant predictor of Phonology [*Estimate* = −8.100, 95% CI = (−15.662, −0.538), *P* = 0.036] and Semantics [*Estimate* = 9.036, 95% CI = (1.249, 16.823), *P* = 0.023], and a marginally significant predictor of Lexical syntax [*Estimate* = 8.777, 95% CI = (−0.501, 18.055), *P* = 0.064].

These were exploratory analyses, not driven by strong hypotheses, and comprising a relatively large number of comparisons, so these connectome disruption results should be interpreted with caution.

## Discussion

This study examined the cognitive and neural basis of fluent speech production deficits in post-stroke aphasia, and how they relate to the effects of overall severity, semantic deficits and phonological deficits. This was done by first combining a comprehensive set of narrative speech production measures (derived from QPA) at lexical, utterance, and sentence levels, with measures of general language impairment and measures of semantic and phonological deficits. These behavioural measures were entered into a bifactor PCA, which extracts a single general factor and a series of orthogonal (uncorrelated) domain-specific factors. The bifactor PCA revealed that QPA measures of sentence/utterance length and complexity loaded primarily on the general severity factor, along with the explicit severity measure of WAB AQ. This factor accounted for 33.1% of the variance in the data. A distinct lexical syntax factor was also detected (accounting for 16.1% of the variance). This factor was comprised primarily of the usage of words that serve a structural/grammatical role rather than a semantic role (closed class words, pronouns, determiners and verbs) and some aspects of sentence planning (proportion of words in sentences, proportion of well-formed sentences). Phonology and Semantics factors had expected loadings (accounting for 11.9% and 9.5% of the variance, respectively) with virtually no contributions from any of the QPA measures, suggesting that QPA measures capture aspects of fluent speech production that are quite distinct from phonological planning and semantic processing.

Prior studies that examined the internal structure of QPA measures^23,55,57^ broadly agree about a lexical–morphological factor (proportions of closed class words, pronouns and verbs) and a sentence-level structural factor (proportion of words in sentences, embedding index, mean sentence and utterance lengths). They disagree about whether correct use of determiners, inflections and auxiliary verbs is morphological–lexical or sentential or a separate grammatical factor, and whether use of ‘well-formed’ sentences belongs with the sentence structure measures or the separate grammatical factor. There is also uncertainty about whether words per minute belongs with the lexical–morphological factor or is a separate factor reflecting speech rate or discourse productivity. The present results converge on the points of agreement (a lexical-level syntax factor and a sentence-level length/complexity factor), but the inclusion of severity measures (WAB AQ and naming accuracy) and prioritizing severity by using a bifactor rotation revealed that length/complexity of speech production is closely related to overall severity. Although several studies have also documented the relationship between fluency and aphasia severity,^35–37,86^ it has not been recognized in studies that did not include explicit measures of severity and/or tested participants in a relatively narrow range of severity.

The ambiguous measures (words per minute; use of determiners, inflections and auxiliary verbs; and well-formedness of sentences) also loaded strongly on this severity-related complexity factor, although words per minute, determiner index and proportion of well-formed sentences also substantively loaded on the lexical syntax factor (as did the proportion of words in sentences). This suggests that the structural complexity of aphasic speech output is strongly related to overall aphasia severity and the separable grammatical factor is largely lexical, corresponding to the correct use of words that primarily serve structural roles and add very little independent semantic content. Production of such semantically ‘light’ words may be particularly dependent on syntactic cues and vulnerable to syntactic deficits,^88^ commensurate with their primarily structural role.

The lesion correlates of these deficit dimensions (factor scores) were then evaluated using SCCAN LSM, a multivariate optimization technique that more accurately identifies symptom-relevant lesion areas, including distributed networks of brain areas. Deficits on the general complexity factor were associated with damage in a fairly large portion of the lesion territory, primarily in parietal and posterior temporal regions. These regions include the dorsal speech production stream^89^ and, in the context of the framework proposed by Matchin and Hickok,^6^ suggest that fluent production of narrative speech—as measured by length and structural complexity of utterances or sentences—is particularly reliant on temporo-parietal regions that support high-level structural planning of longer utterances and more complex sentence structures. However, there was a substantial overlap between this factor and overall aphasia severity (WAB AQ), both in the PCA results and in the SCCAN LSM results. Both the complexity factor and aphasia severity were associated with damage throughout the lesion territory, even extending dorsally beyond traditional language areas, reflecting that damage most anywhere in the left MCA territory can produce an aphasia-relevant deficit (as we have previously found^48^). This suggests that a broad range of different language deficits can contribute to the overall aphasia severity score and to the length and complexity of aphasic narrative speech.

Deficits on the lexical syntax factor were associated with damage to a small set of fronto-parietal regions: dorsal inferior parietal lobule (dIPL), precentral gyrus, and IFG pars triangularis. Precentral gyrus and dIPL are not regions that previous studies have consistently associated with syntactic deficits or agrammatism, which are more typically associated with posterior temporal and/or inferior frontal damage.^6^ IFG is associated with a broad range of language processes (including syntax) and is the subject of ongoing debate. Because the lexical syntax factor was orthogonal to the complexity factor, it is unlikely that the IFG involvement observed here reflects syntactic planning, and because the factor is also orthogonal to the Phonology factor, it is unlikely that this reflects phonological–articulatory planning. A remaining possibility is that the IFG involvement reflects the recruitment of control systems to support the difficult retrieval of semantically ‘light’ words,^87^ which primarily serve syntactic roles and do not have strong support from semantic representations.^88^

Tract-based measures of connectivity disruption were weakly (if at all) associated with the deficit scores after controlling for overall lesion volume. In part, this may be because atlas-based estimation of tract damage is not very effective.^90^ Even if the tract damage measures were very good, tract disconnection and behavioural deficit measures were both strongly correlated with overall lesion volume, leaving relatively little variance for an association between tract disconnection and behavioural deficit.

The graph theoretical measures of connectivity produced a few suggestive patterns. First, measures derived from a left hemisphere peri-Sylvian language network were better predictors than the same measures calculated based on the connectivity of the whole brain. This may be because connectivity within the language network is particularly important for residual language function after left hemisphere stroke (the present data do not address whether this is also true in neurologically intact individuals) or because this is the region where there was the greatest variability between participants, since this is where most of them had lesions (as is normally the case for post-stroke aphasia).

Second, within the language network, global efficiency was the most consistent predictor, being significantly associated with each of the four PCA-derived deficit measures. Global network efficiency is high when the paths between nodes are relatively short and the nodes can exchange information relatively quickly and efficiently^31,80^ (it is also known as propagation speed^32^ or propagation steps^91^). In this case, higher efficiency scores mean that the left hemisphere language network is more functionally integrated and the results indicate better integration of the left hemisphere language network is positively associated with length and complexity of aphasic speech output, its lexical–syntactic content, and reduced phonological deficits (better phonological ability). Surprisingly, the efficiency of the left hemisphere language network was *negatively* associated with semantic ability. Semantic cognition relies on a bilateral ‘hub-and-spoke’ architecture^92^ and the global efficiency measure tends to be less useful for larger and sparser networks,^31^ so these results should be interpreted with caution.

Third, the average clustering coefficient was significantly positively associated with performance on phonological and semantic measures (and marginally with lexical syntax). This suggests that phonological and semantic processing (and, somewhat less reliably, syntax) are supported by relatively specialized, densely connected clusters or sub-networks, as captured by the clustering coefficient measure.

### Limitations

Several key limitations need to be considered when drawing conclusions from the present results. The participant sample was limited by the availability of narrative speech samples and lesion data. This resulted in a sample size (*N* = 58) that was moderate relative to other LSM studies, with most of the participants in the mild-to-moderate range of aphasia severity (median WAB AQ = 81.0, IQR = 71.3–91.0), though it did include some participants with more severe aphasia. Similarly, although the sample included participants across nearly the full range of fluency, most participants were in the upper half of the range (median WAB fluency = 8.0, IQR = 5.0–9.0, range = 2.0–10.0), which is traditionally considered ‘fluent’ aphasia, though individuals in this range nevertheless typically have some disruptions to fluency. This severity distribution is a natural consequence of requiring at least 150 narrative words for QPA scoring—very severely impaired participants are often unable to produce that many narrative words, especially if their impairment is severely non-fluent. Both PCA and LSM are most effective when the sample covers a broad range of severity, including participants with very mild (possibly sub-clinical) deficits. Therefore, the present results are informative about fluency deficits in mild-to-moderate post-stroke aphasia, but it is possible that the pattern of deficits in the severe range is somewhat different.

The behavioural data were adequate for PCA according to standard metrics (KMO > 0.7), but the PCA results need to be considered in the context of which behavioural measures were included and not included (or not available). We sought to balance detailed measures of narrative speech with key anchors (aphasia severity, semantic deficit, phonological deficit). The present study included fewer grammatical measures than prior PCA studies of QPA and fluency^35,37,57^ but compared with other PCA studies of aphasia,^39,41^ it included more grammatical measures and fewer other measures. This fills an important gap in the literature by identifying how aspects of connected speech production are related to or independent of those key anchors. Specifically, compared with PCA studies that focused on QPA, we observed fewer QPA-specific factors, largely because measures of speech length and complexity were strongly related to overall aphasia severity rather than forming separate factors. We did find a lexical syntax factor, which is common for QPA-focused studies but rare for broad PCA studies of aphasia, suggesting that this is a distinct dimension of impairment. Measures of motor speech impairment (dysarthria or apraxia of speech) and executive function deficits were not available for inclusion in the present study, so the results do not address how the observed patterns relate to those deficits.

Another key limitation is that diffusion data were not available for the participants, so connectivity disruption was estimated by overlaying each participant’s lesion map on a tractography atlas derived from the HCP-842 data. This method has been widely used, but (to our knowledge) has not been validated and we have previously expressed caution about using it.^90^ We found that, after controlling for overall lesion volume, tract-based connectivity disruption was weakly (if at all) associated with the deficit scores and graph theory measures of connectivity disruption produced very mixed and inconsistent results. Although unsatisfying on their own, we hope these results will encourage further development and validation of connectivity disruption measures to quantify this important aspect of brain injury.

## Summary and conclusion

In sum, the present results demonstrate that length and complexity of narrative speech production is closely related to severity, both in the behavioural sense (i.e. aphasia severity as measured by WAB AQ) and in the neural sense (a large portion of the left MCA territory is associated with reduced length and complexity of narrative speech production). In contrast, lexical aspects of syntax (usage of closed class words, pronouns, verbs, and determiners) appear to be separable from the severity and associated with damage in a smaller set of fronto-parietal regions, suggesting that these deficits may be related to the impaired requirement of control systems to support retrieval and correct usage of words that primarily serve structural functions rather than adding semantic content. Finally, graph theoretic measures of language network integration (efficiency) and cohesion of sub-networks (clustering coefficient) were statistically associated with the deficit scores after controlling for lesion volume, suggesting that these measures of connectivity deserve further investigation.

## Supplementary Material

fcac327_Supplementary_Data

## Data Availability

Anonymized behavioural data and lesion data are available from Daniel Mirman (dan@danmirman.org) subject to approval by the Einstein Healthcare Network Institutional Review Board.

## Abbreviations

AF =: arcuate fasciculus
AQ =: aphasia quotient
CCT =: Camel and Cactus Test
CI =: confidence interval
CV =: cross-validation
FAT =: frontal aslant tract
IFG =: inferior frontal gyrus
IFOF =: inferior fronto-occipital fasciculus
KMO =: Kaiser–Meyer–Olkin
LSM =: lesion–symptom mapping
MCA =: middle cerebral artery
PCA =: principal component analysis
PNT =: Philadelphica Naming Test
PRT =: Philadelphia Repetition Test
QPA =: Quantitative Production Analysis
SCCAN =: sparse canonical correlation analysis for neuroimaging
SLF =: superior longitudinal fasciculus
SMG =: supramarginal gyrus
UF =: uncinate fasciculus
VP =: verb phrase
WAB =: Western Aphasia Battery
