# Supplementary material for: Behavioural and neural structure of fluent speech production deficits in aphasia
Source: Brain Commun. 2022 Dec 14;5(1):fcac327. doi: 10.1093/braincomms/fcac327 (PMC9798301; doi:10.1093/braincomms/fcac327)
Supplement: fcac327_Supplementary_Data [file fcac327_supplementary_data.pdf]

Supplementary material

Supplementary Figure 1: Bivariate correlations among behavioural measures.

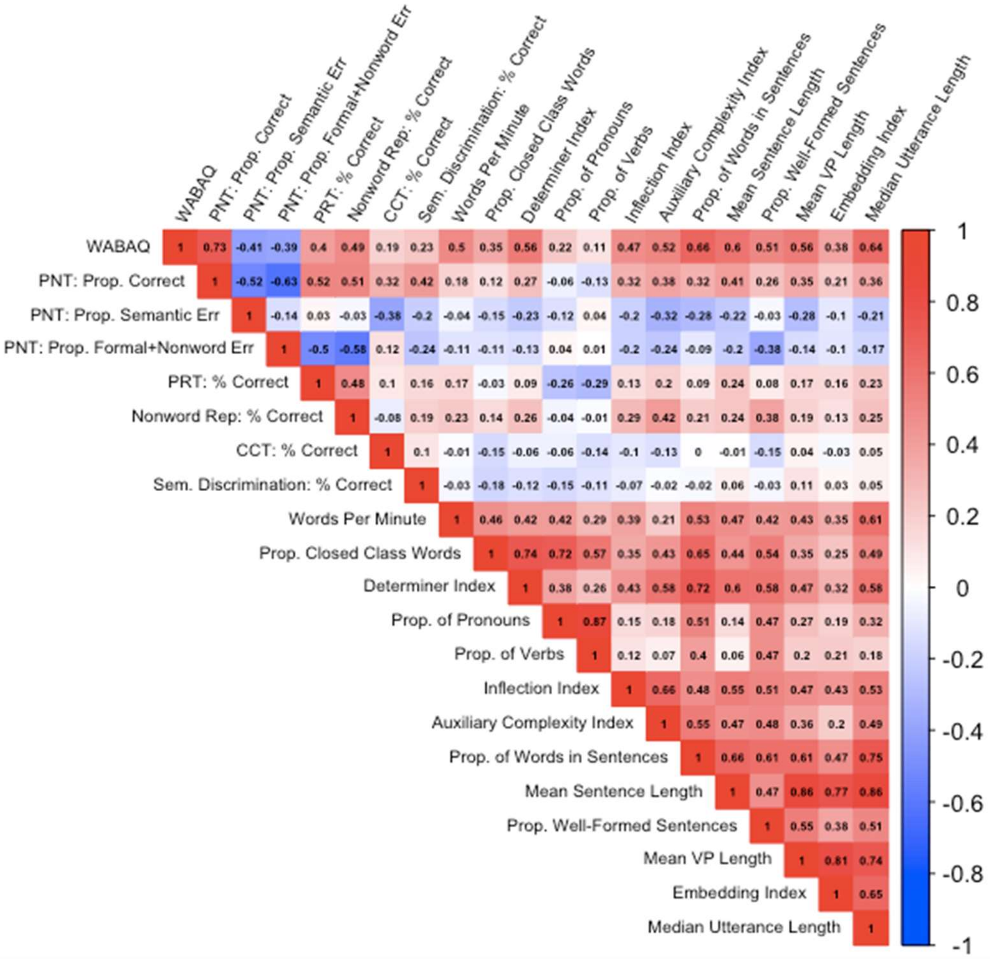

Supplementary Figure 2: Lesion overlap map (at MNI coordinates, from left to right: x = -36, z = 19, y = 3). Colours show number of participants with a lesion in each voxel. Only voxels with >10% (n=6) lesion involvement were included in analyses.

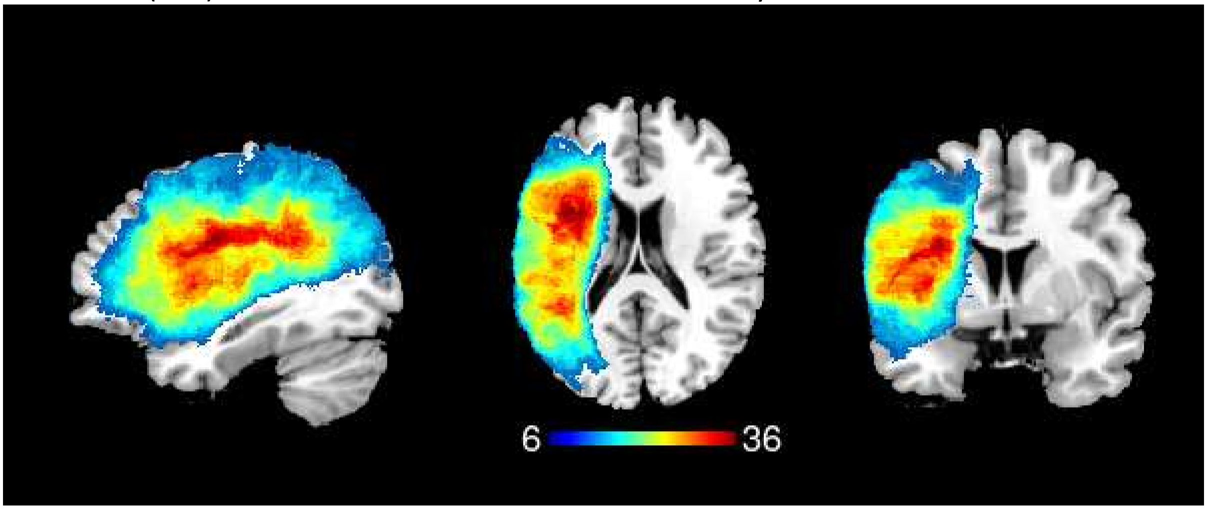

Supplementary Table 1. Summary of QPA measures

QPA measures	Formula	Definition (based on Saffran et al., 1989)
**Words per minute**	Total words produced / Total number of minutes	Measure of speech rate
**Proportion of closed class words**	(Total narrative words – Open Class words) / Total narrative words	Closed class words: words that are not nouns, verbs, adjectives and adverbs (open class words)
**Determiner index**	Nouns with a required determiner / Nouns requiring a determiner	Proportion of nouns requiring determiners (proper nouns and plural forms might not require determiners depending on the context) that were produced with a determiner.
**Proportion of pronouns**	Pronouns / (Nouns + Pronouns)	Only personal pronouns are scored.
**Proportion of verbs**	Verbs / (Nouns + Verbs)	Verbs: all verb forms (infinitive, gerundive etc.), not limited to the main verb
**Inflection index**	Inflectable verbs that were inflected / Inflectable verbs	Verbs in inflected form (-s, -ies, -ed, -ied, -ing)
**Auxiliary complexity index**	(Total auxiliary score / Matrix verbs) - 1	Matrix verb: the main verb of an independent clause. Each auxiliary element of the matrix verb is assigned a score of +1, increasing the verb's score from the baseline of 1.
**Proportion of words in sentences**	Words in sentences / Total narrative words	Narrative words: all words that reflect the propositional speech produced as part of the narrative.<br>Sentences: defined as an utterance following one of the following forms: (a) noun + main verb, (b) noun + copula + adjective, or (c) noun + copula + prepositional phrase
**Mean sentence length**	Words in sentences / Total sentences	
**Proportion of well-formed sentences**	Well-formed sentences / Total sentences	Well-formed sentences: sentences requiring at least a verb and a noun
**Mean VP length**	Words in Verb Phrases / Verb Phrases	Words in VP phrases: the sum of open class words + pronouns within the VP phrases.
**Embedding index**	Embeddings / Total sentences	The number of embedded clauses per sentence
**Median Utterance Length**	Median length of all utterances	Utterances refer to a sum of narrative words that forms a coherent, independent unit. These can be sentences, topic/comment statements, isolated narrative words or phrases, etc. Prosodic and syntactic indicators are considered for the segmentation of a speech sample into utterances.

Supplementary Table 2. Bifactor PCA factor loadings.

Measures	RC1 (Complexity)	RC2 (Lexical Syntax)	RC4 (Phonology)	RC3 (Semantics)
**Mean Sentence Length**	0.852	0.025	0.427	-0.028
**Median Utterance Length**	0.797	0.204	0.362	0.025
**WAB AQ**	0.785	0.175	-0.083	0.385
**Mean VP Length**	0.763	0.095	0.444	0.035
**Inflection Index**	0.689	0.114	0.115	-0.105
**Naming Accuracy (PNT)**	0.663	-0.133	-0.371	0.545
**Proportion Words in Sentences**	0.654	0.523	0.268	0.111
**Embedding Index**	0.648	-0.002	0.486	-0.110
**Nonword Repetition**	0.636	-0.037	-0.627	-0.043
**Aux. Complexity Index**	0.636	0.214	-0.027	0.015
**Proportion Well-Formed Sentences**	0.624	0.503	-0.086	-0.190
**Determiner Index**	0.594	0.489	0.167	0.001
**Phonological Errors (PNT)**	-0.538	0.085	0.695	0.132
**Word Repetition (PRT)**	0.530	-0.202	-0.682	-0.025
**Proportion Verbs**	0.018	0.865	-0.004	-0.075
**Proportion Closed Class Words**	0.397	0.778	0.059	-0.060
**Proportion Pronouns**	0.090	0.926	0.070	0.046
**Words per Minute**	0.509	0.381	0.123	-0.020
**Semantic Discrimination**	0.193	-0.179	-0.273	0.532
**Semantic Errors (PNT)**	-0.224	-0.082	-0.183	-0.773
**Camel and Cactus Test**	-0.033	-0.116	0.097	0.749
**SS Loadings (Eigenvalues)**	6.956	3.381	2.496	1.992
**Proportion of Variance**	0.331	0.161	0.119	0.095
**Cumulative Variance**	0.331	0.492	0.611	0.706

Supplementary Table 3. Spearman rank correlations between connectivity disruption measures and each behavioural measure. Overall lesion volume is included in both to show its relationship to behavioural measures as well as to measures of connectivity disruption.

	Volume	Complexity	Lexical Syntax	Phonology	Semantics
**Volume**	-	-0.47 **	-0.331 *	-0.216	-0.244 .
**Tracts**					
AF	0.545 **	-0.316 *	-0.311 *	0.0445	0.0064
FAT	0.683 **	-0.366 **	-0.292 *	-0.217	-0.217
IFOF	0.543 **	-0.142	-0.020	-0.240 .	-0.250 .
SLF	0.736 **	-0.459 **	-0.359 **	-0.163	-0.00687
UF	0.527 **	-0.166	-0.0595	-0.204	-0.253 .
**Whole-Brain Network**					
Characteristic path length	-0.128	-0.0656	-0.0651	0.0318	-0.00483
Ave Clustering coefficient	0.322 *	-0.0236	-0.0899	-0.0596	0.0203
Global efficiency	0.0392	0.0742	0.0581	-0.0272	0.0367
**Language Network**					
Characteristic path length	0.566 **	-0.36 **	-0.278 *	-0.148	-0.0863
Ave clustering coefficient	-0.0247	-0.0749	0.152	-0.118	0.219 .
Global efficiency	0.0683	0.0166	0.136	-0.204	0.0272

Note: ** $p < 0.01$, * $p < 0.05$, . $p < 0.1$

Supplementary Analysis: Aphasia Severity (WAB AQ)
Because the first factor, Complexity, was closely related to aphasia severity, a comparison SCCAN LSM was conducted for WAB AQ alone, not combined with other measures as part of the first factor. WAB AQ score was associated with damage to a very similar (even larger) portion of the MCA territory (optimal sparseness = 0.798, CV correlation = 0.653, $p < 0.001$) that included parietal, temporal, and frontal regions (Supplemental Figure 3). The primary differences were that the lesion correlates of WAB AQ score extended more anteriorly into frontal regions (precentral gyrus, IFG, MFG), posteriorly into angular gyrus, and inferiorly into middle temporal gyrus.

Supplementary Figure 3. SCCAN LSM results for aphasia severity. The colours correspond to normalised SCCAN weights in the range 0-1. Results are shown on slices of an MNI template (from left to right: x = -53, -50, -45, -36, and z = 11).

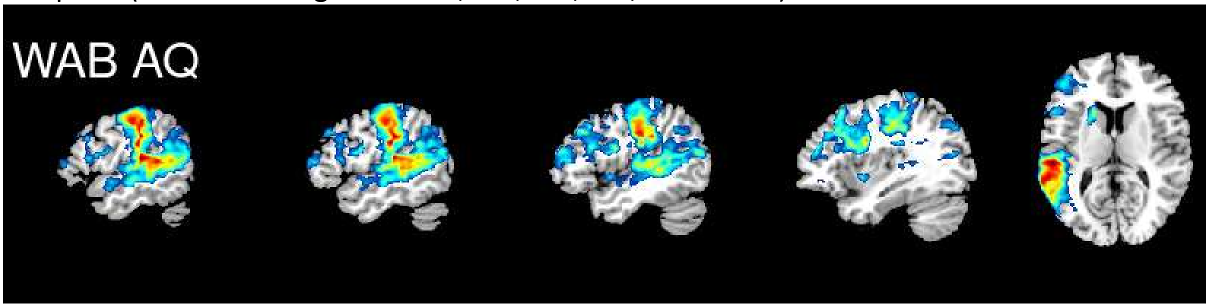

In regression analyses of connectivity disruption, only lesion volume was a statistically significant predictor of WAB AQ (Supplementary Table 4). Neither tract-based nor graph theory measures of connectivity disruption accounted for significant variance in WAB AQ after lesion volume was taken into account.

Supplementary Table 4. Results of multiple regression with robust estimation of standard errors for connectivity disruption predictors of WAB AQ. Values show the regression coefficient estimate [95% confidence interval in brackets].

**Tracts**		
Volume	-0.10 [-0.14, -0.062] **	
Arcuate fasciculus	-0.028 [-0.15, 0.089]	
Frontal aslant tract	-0.052 [-0.13, 0.026]	
Superior longitudinal	0.031 [-0.1, 0.17]	
Uncinate fasciculus	0.057 [-0.068, 0.18]	
Inferior fronto-occipital	0.028 [-0.081, 0.14]	
**Graph theory measures**	**Whole-brain network**	**Language network**
Volume	-0.11 [-0.15, -0.067] **	-0.0072 [-0.011, -0.0037] **
Global efficiency	-860 [-3000, 1300]	-13 [-200, 170]
Ave clustering coefficient	3800 [-2100, 9700]	150 [-390, 700]
Characteristic path length	0.014 [-0.0084, 0.037]	0.00070 [-0.0013, 0.0027]
